# Application of a Generalized Utility Function to Determine the Optimal Composition of Geopolymer Mortar

**DOI:** 10.3390/ma17246237

**Published:** 2024-12-20

**Authors:** Maja Kępniak, Faustyn Recha, Piotr Prochoń

**Affiliations:** 1 Department of Building Materials Engineering, Faculty of Civil Engineering, Warsaw University of Technology, Armii Ludowej 16, 00-637 Warsaw, Poland; piotr.prochon@pw.edu.pl; 2 Faculty of Architecture, Civil Engineering and Applied Arts, Academy of Silesia, Rolna 43, 40-555 Katowice, Poland; faustyn.recha@wst.pl

**Keywords:** geopolymer, generalized utility function, optimal composition, perlite powder

## Abstract

The aim of the presented research was to evaluate the impacts of modifications to the technical properties of fly ash-based geopolymer composites, particularly focusing on enhancing the thermal insulation. Through the utilization of a generalized utility function, optimal dosages of additives such as perlite sand, waste perlite powder, and cenospheres were determined. The study aimed to increase the thermal insulation of the composites while maintaining satisfactory compressive and flexural strength. The results indicated that dosages of perlite dust and cenospheres significantly influenced the technical characteristics of the composites; an exception was the flexural strength, for which these modifications did not show a statistically significant effect. The average compressive strength values, except for the mixes with poor workability, were at least 3.5 MPa (RILEM class II). Notably, a balanced dosage of additives, around 75 kg per cubic meter of the mixture in the total mixture, yielded the most favorable outcomes in terms of thermal isolation (0.18–0.24 W/(m·K) and workability (cone immersion 40–70 mm). Additionally, perlite dust emerged as a potentially superior modifier due to its waste origin. However, further analysis considering life cycle parameters including the carbon footprint and water footprint would be necessary to validate this claim. Overall, the study highlights the potential of utilizing perlite-based modifiers to enhance the thermal insulation of geopolymers while addressing environmental concerns.

## 1. Introduction

Concrete, a ubiquitous building material [1], contributes significantly to carbon emissions and energy consumption during its production process [2]. The emergence of geopolymers as alternative binders presents a promising avenue for addressing these environmental concerns. Geopolymers, characterized by their amorphous, inorganic structure primarily comprising calcium–aluminum–silicon compounds [3], offer superior properties such as rapid strength gain [4]; high mechanical strength [5]; and exceptional resistance to fire [6], sulfate [7], and acid attacks [8]. Moreover, their production entails lower carbon dioxide emissions and energy consumption compared with conventional cement [9,10]. The advancement of geopolymer materials presents a significant opportunity to enhance the sustainability and application potential of construction materials. Geopolymer materials find versatile applications across various industries, including cement matrix, high-temperature-resistant materials, thermal insulation, and even the production of lightweight fire-resistant concretes and coatings. Despite the widespread application of geopolymers in various construction industries, there remains a research gap concerning their utilization in shotcrete technology. While geopolymers have been employed in diverse applications such as repair materials [11], marine corrosion-resistant materials [12], solidified waste materials [13], and crack grouting materials [14], their potential as thermal isolating mortars remains largely unexplored. Addressing this gap necessitates further research and development to optimize geopolymer materials for isolating applications, thereby expanding their scope within the construction industry.

The concept of a generalized utility function [15] has gained prominence in various engineering disciplines, including the sustainable building design [16]. A generalized utility function serves as a mathematical framework to evaluate and optimize the performance of composite materials based on multiple criteria, such as mechanical properties, durability, cost-effectiveness, and sustainability factors [17]. In the realm of building composites, where the demand for multifunctional materials is growing, the application of a generalized utility function offers a systematic approach to address complex design challenges. The application of a generalized utility function in the case of building composites allows for determining the optimal composition of the mixture while considering that a modification may positively impact one characteristic while negatively affecting others. It also enables the identification of optimal compositions by not favoring over-engineered blends, for example, those with compressive strength higher than required [18]. The application of this function also allows for considering the significance of specific characteristics. For instance, in structural applications, mechanical properties such as strength, thermal insulation, and ductility may be of paramount importance, while in architectural applications and aesthetic considerations, other properties may take precedence [19]. Moreover, the multicriteria analysis enables the consideration of life cycle assessments (LCAs) and environmental impact analyses in the composite design process [19]. By quantifying the environmental footprint of materials throughout their life cycle, engineers can make informed decisions to minimize resource consumption, energy use, and greenhouse gas emissions associated with building composites [20]. A generalized utility function offers a systematic and holistic approach to designing building composites that meet diverse performance criteria while considering sustainability and cost-effectiveness by integrating multidimensional analyses and advanced mathematical technique techniques such as neural networks [21], genetic algorithms [22], and generalized additive models [23] to search for solutions that balance conflicting objectives and constraints.

The aim of the presented research was to utilize the generalized utility function to determine the optimal dosage of additives potentially enhancing the thermal insulation of fly ash-based geopolymer composites. The conducted modifications aimed at increasing the thermal insulation of the composite without significant adverse effects on compressive and flexural strength. The modifiers used in the study included perlite sand, waste perlite dust, and cenospheres.

## 2. Materials and Methods

The research investigated the potential enhancement of thermal insulation in geopolymer composite by two additives: perlite powder (PP, JAWAR, Ciechanów, Poland), and cenospheres (C, Aliorsun, Piaseczno, Poland). The objective was to determine the optimal dosage ranges of each component that would achieve good thermal insulation, good compressive strength, and sufficient workability. The composition of 1 m^3^ of the analyzed mortar consisted of 500 kg of precursor mix, comprising 80% fly ash (Siekierki CHP Plant, Warszawa, Poland), 10% metakaolin (Astra, Straszyn, Poland), and 10% zeolite (Astra, Straszyn, Poland); 315 kg of alkaline activators; 30% sodium hydroxide (PCC Group, Brzeg Dolny, Poland) and water glass (Zakłady Chemiczne Rudniki S.A., Rudniki, Poland); 110 kg of calcium formate (Warchem, Zakręt, Poland); and 15 kg of rheology modifiers (ELOTEX FX 2320, Celanese, Sempach Station, Switzerland). The investigations of the chemical compositions, mineral phase characterizations, and physical characteristics of the components have been described in preceding articles [24,25]. The dosage of perlite sand (PS, JAWAR, Ciechanów, Poland) was determined in earlier stages of the experiment and remained constant at 30 kg [26]. Perlite powder and cenospheres were varied according to the two-factor experimental design. The factor analysis focused on two parameters of composite composition: the quantity of perlite powder and cenospheres per 1 m^3^. Preliminary studies established upper levels, with 75 kg/m^3^ adopted for both perlite powder and cenospheres. To ensure consistency in the composition of the mixtures, a minimum dosage of 5 kg/m^3^ of mixture was adopted for both perlite powder and cenospheres. The amount of natural sand required for 1 m^3^ of the mixture was determined for each trial. To simplify the statistical analysis, a two-factor experimental design was used, with measurements taken twice at the central point for accuracy. The statistical name of the plan was two-factor rotational central composite design (CCD) [27]. The complete design and variable levels are detailed in Table 1, while the figure in Table 1 illustrates the distribution of the design points in the factor space.

In this research, the consistency test was performed on a 10 L mixture by the immersion of a 300 g cone (slant height 150 mm, max. diameter 75 mm) in 10 s. The undesirable characteristic observed in the composite, indicating a critical interaction among dependent variables, was a thermal conductivity exceeding 0.75 W/(m·K), RILEM class II [28]. The experimentation involved slab-shaped specimens measuring approximately 40 mm thick and 300 mm by 300 mm in dimensions. Utilizing the FOX 314 (TA Instruments, New Castle, DE, USA) plate apparatus, thermal conductivity measurements were conducted under stabilized conditions employing the “hot plate” method. The upper and lower plates maintained a temperature difference of 20 K, while the sample temperature was set at 10 °C. To assess the flexural strength of the composite, three rectangular specimens, each measuring 40 mm by 40 mm by 160 mm, were fabricated for every composition. These specimens underwent testing in accordance with the EN 196-1 [29] standard, employing the three-point loading method. For evaluating the compressive strength of the composite, six specimens were prepared for each composition, also adhering to the EN 196-1 [29] standard. The compressive area of each specimen was 1600 mm^2^.

To create a material model of modified geopolymer and to prepare a generalized utility function, the following procedure was employed:

- Independent variables were identified: the level of perlite powder and the level of cenosphares.
- The range of variability of independent variables was determination.
- Dependent variables in the form of analyzed material characteristics were identified.
- An optimal experimental plan was developed.
- Concrete mixes and concretes were prepared, followed by measurements of dependent variables.
- Based on the obtained results of laboratory tests, equations connecting the value of dependent variables with independent variables were found.
- The calculated equations—the selection of the simplest dependencies meeting the requirements imposed on regression equations—were verified and assessed based on the mean absolute percentage error (MAPE), $R^{2}$ coefficient, and analysis of correlation and partial autocorrelation of residuals.
- The set of verified regression equations constituted the material model.
- For selected key material characteristics, worse and better values were assumed, and based on these, partial values of generalized utility were calculated.
- Specific weights were assigned to selected characteristics, and the values of generalized utility U were calculated.
  Based on the obtained values of the utility function U, a regression equation describing it was established.

One of the successfully utilized tools in civil engineering materials is the creation of material models. A material model represents a mathematical relationship between composition (independent variables) and properties (dependent variables). This means that the model establishes connections between the variables defining the composition, such as the content of a particular component, and the measurable properties of the composite, such as its strength or thermal conductivity, which depend on the composition. In other words, the model predicts how changes in the material’s composition affect its overall properties. The model, considering the time factor, is termed a dynamic model, while excluding this factor, it is termed a static model. In this work a static model was employed, except for the carbonation process analysis, where time was considered as an independent variable.

Due to the non-homogeneity of cement concrete, a definitive relationship between composition and properties cannot be defined. The characteristics of such a material are considered random variables. Therefore, established composition–property relationships have a regression, not deterministic, nature. The sought material model in this work was statistical–experimental as it was constructed based on planned experiments and statistical analysis of their results. The development of the material model for concrete modified with the studied waste was based on statistical experimental design. This approach was justified by minimizing the required trials while allowing the attainment of regression functions with a high correlation coefficient. In order to utilize the statistical experimental design, material variables were presented in a standardized form. They were introduced according to the following rule (1):

(1) $$x_{cod}=\frac{x_{real}-x_{0}}{0.5\cdot ∆x}\to x_{real}=x_{cod}\cdot 0.5\cdot ∆x+x_{0}$$

where $x_{cod}$ is the standardized (coded) variable, $x_{real}$ is the real variable, $x_{0}$ is the midpoint of the variability range, and ∆x is the range of variability.

The range ∆x constitutes the experiment area for a given material parameter, and for the standardized variable, it assumes values in the range $\langle -1.414;1.414\rangle$. The obtained material model in the study was based on the results obtained from statistical experimental design and the regression functions determined based on them, along with appropriate correlation coefficients and calculated mean errors, as well as autocorrelation and partial autocorrelation functions. To find the regression equations, the method of generalized additive models (GAM) was employed. This was motivated by variance non-uniformity, linear correlation, and the distribution of some variables being non-normal. Generalized additive models are an extension of techniques developed and popularized by Hastie and Tibshirani [30]. Detailed descriptions of these techniques, algorithms used for model fitting, and research discussions in this area of statistical modeling can also be found in Schimek’s publication [31]. Generalized additive models are an extension and expansion of generalized linear models. In the case of generalized linear models, the values of the transformed dependent variable are predicted based on a linear combination of independent variables. The transformation is determined by the linking function. Different distributions of the dependent variable can be assumed. In the case of generalized additive models, instead of a linear combination of independent variables, a non-parametric function obtained by smoothing the scatter plot of partial residuals is used. In the generalized additive model, it is assumed that the relationship between variables has the following form (2) [26]:

(2) $$Y=g\left(b_{0}+{b_{1}X}_{1}+{b_{2}X}_{2}+\ldots +{b_{k}X}_{k}\right)+e$$

Formally, the inverse function to $g\left(\ldots \right)$, denoted as $f\left(\ldots \right)$, is called the linking function, such that (3) [27]

(3) $$f\left(m_{i.y}\right)=b_{0}+{b_{1}X}_{1}+{b_{2}X}_{2}+\ldots +{b_{k}X}_{k}$$

where $g\left(\ldots \right)$ is a certain function; e represents the error level; $m_{i.y}$ denotes the expected value of y; and coefficients $b_{0},b_{1},\ldots ,b_{k}$ correspond to the independent variables $X_{0},X_{1},\ldots ,X_{k}$.

Depending on the assumed distribution of variable Y, different linking functions can be used. The simplest linking functions are the identity, logarithmic, and power functions. To perform calculations and find regression equations, the Statistica 13 program (StatSoft Europe GmbH) was utilized.

In order for the obtained mathematical models to be recognized as regression equations, they must be verified by assessing the convergence of the results obtained from the model with empirically obtained values. The measures of conformity are the correlation coefficient R and determination $R^{2}$. The R coefficient characterizes the strength of the relationship between the independent and dependent variables of the model [32]. The $R^{2}$ coefficient is a measure of the model’s fit quality. Suggested fit measurements for building composites are as follows [33]: a well- or very well-fitted model $R^{2}$ ≥ 0.75 and satisfactorily consistent $R^{2}$ ≥ 0.65. To determine the correctness of the model or choose one of several considered fits, calculations of the MAPE error (4) were used. Generally, the following is accepted when MAPE takes the following values [34]:

- MAPE ≤ 3%, the forecasts are highly accurate;
- 3% < MAPE ≤ 5%, the forecasts are considered good;
- 5% < MAPE ≤ 10%, the forecasts are somewhat accurate but acceptable;
- MAPE > 10%, the forecasts are inaccurate and should not be accepted.

For the final verification of the obtained equations, the autocorrelation functions and partial autocorrelation functions of the residuals were checked, and it was assessed whether they fell within acceptable limits.

The concept of the generalized utility function, developed by Harrington [35], enabled the determination of a generalized multi-criteria model for concrete modified with mineral waste dust. Thanks to this generalized model, it was possible to assess the overall quality of the resulting composite [17,36,37]. The values of individual properties, expressed using the obtained regression equations, were scaled dimensionlessly. They were assigned distributions in the form of lower, intermediate, and upper values, and corresponding utility values were assigned to them. For properties where a maximum criterion applied, a utility value of 1.0 was assigned to the upper values, and for properties where a minimum criterion applied, a utility value of 1.0 was assigned. For properties with a maximum criterion, the highest values received the highest utility, while for properties with a minimum criterion, the lowest values received the highest utility, and intermediate values were assigned utility values proportionally decreasing with their deviation from the extreme. The regression equations and variable significance analyses were performed using the Statistica 13 (StatSoft Europe GmbH, Hamburg, Germany) software, while the calculations of the generalized utility function were carried out outside the software by the authors. To calculate the generalized utility function, Equation (4) [35] was utilized:

(4) $$U=exp\left[-\sum_{i=1}^{m}w_{i}exp\left(-\frac{y_{i}-y_{iw}}{y_{ib}-y_{iw}}\right)\right]\;for:0\leq w_{i}\leq 1;i=1,2,\ldots .,m;\sum_{i=1}^{m}w_{i}=1$$

where $w_{i}$ is the weight of the feature, $y_{i}$ is value of the feature, $y_{iw}$ is lower boundary of the satisfying range of the feature (worse value), and $y_{ib}$ is upper boundary of the satisfying range of the feature (better value).

A thermal field emission scanning electron microscope (FE-SEM, Hitachi, Tokyo, Japan) was used to investigate the morphology of the geopolymer mortars. The SEM images were captured for mortars with composition 7 at magnifications of (a) 1000× and (b) 5000×. Mortar samples were obtained from beam specimens measuring 4 cm × 4 cm × 16 cm. These beams were sectioned into 1 cm thick slices, dried, and prepared for SEM analysis by coating them with a thin gold layer under vacuum conditions. This methodology for examining mortars has already been successfully applied in similar studies [37,38,39,40].

## 3. Results

The prepared composites were tested according to the experimental plan for consistency, flexural strength, compressive strength, and thermal conductivity. The results are summarized in Table 2. Each composition immediately after mixing had sufficient workability to effectively prepare samples. The flexural strength ranged between 1.1 MPa and 2.0 MPa. The compressive strength ranged from 1.9 MPa to 6.9 MPa. The thermal conductivity coefficient for all composites was below 0.3 W/(mK), indicating their good thermal insulation properties. The results related to consistency did not have standard deviations because the samples were made from a single composite mix, and therefore, there was only one measurement result.

For all analyzed dependent variables: consistency, flexural strength, compressive strength, and thermal conductivity coefficient, basic statistical analyses were conducted. According to the Shapiro–Wilk test conditions, all variables exhibited a normal distribution (Table 3). The *p*-value for the Shapiro–Wilk test was used to assess the normality of the data. A *p*-value greater than 0.05 indicated that the data likely followed a normal distribution, while a *p*-value of 0.05 or less suggested that the data did not follow a normal distribution.

### 3.1. Microstructure

**Figure 1:**
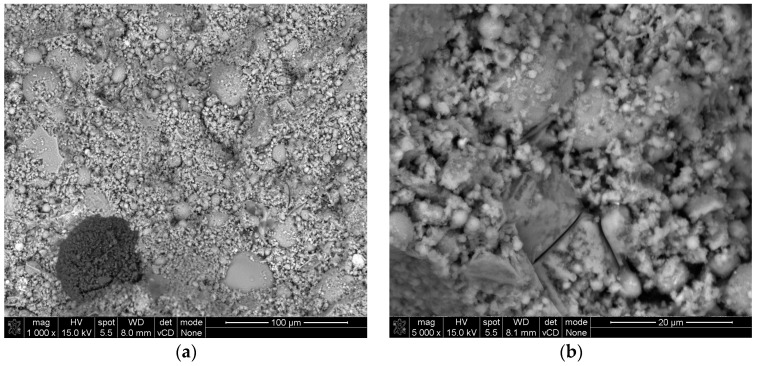
SEM pictures of unmodified geopolymer mortars: (**a**) 1000× magnification and (**b**) 5000× magnification.

**Figure 2:**
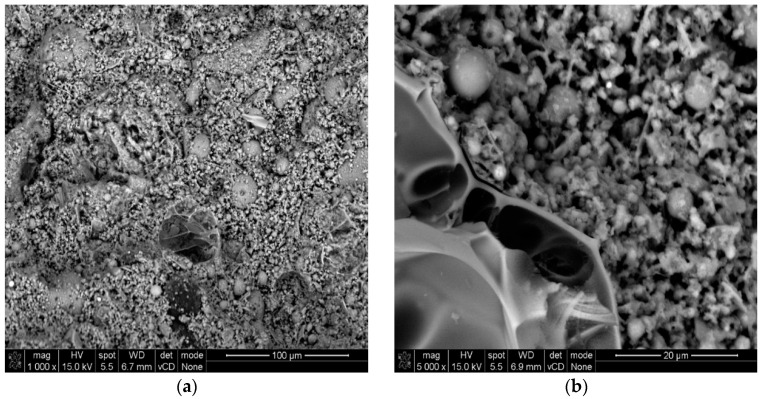
SEM pictures of geopolymer mortars with the composition 7: (**a**) 1000× magnification and (**b**) 5000× magnification.

Unmodified geopolymers tend to form a more visually homogenous gel microstructure with a denser, less porous matrix and a less visible interfacial transition zone between the gel phase and the aggregate (Figure 1). In contrast, composition no. 7, selected as the optimal mixture based on all analyzed parameters, exhibited a microstructure that was porous and abundant in voids between particles of cenospheres, perlite sand, and perlite powder. As shown in Figure 2 this porous microstructure was largely due to the increased use of air-entraining additives in place of natural sand, which significantly influenced its overall structure.

### 3.2. Consistency

As a result of the conducted analyses, functions describing the relationships between independent and dependent variables were identified. For the obtained consistency measurements results $\alpha_{con}$, a GAM analysis was conducted. As a result, Equation (5) was obtained. For the obtained equation, goodness-of-fit checks were performed: MAPE was 9.1%, and *R*^2^ was 0.91. The autocorrelation and partial autocorrelation functions of the residuals were within acceptable ranges, indicating that the obtained equation was a regression equation, and therefore, it could be utilized for further analysis.

(5) $$\alpha_{con}=254.1-3.879PP-3.991C+0.0172{PP}^{2}+0.051PP\cdot C+0.0158C^{2}$$

**Figure 3:**
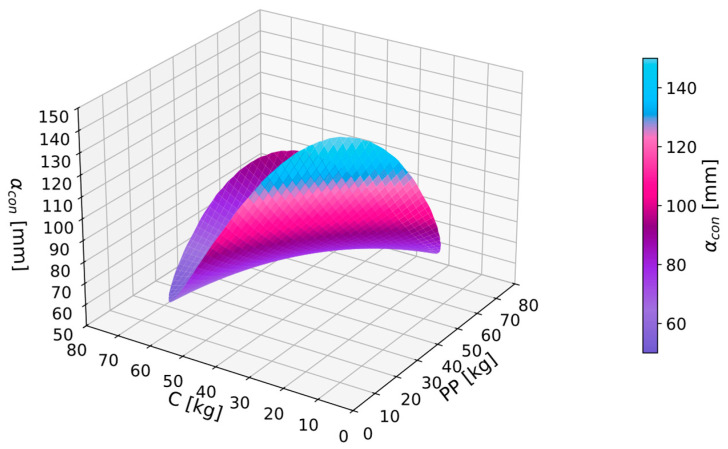
GAM function for consistency $\alpha_{con}$ in dependence of perlite powder PP and cenosphares C.

It can be observed that with an increase in the proportion of components such as perlite powder and cenospheres, the immersion depth of the measuring cone decreased. Workability was poorer, and there was a risk of insufficient compaction of the fresh composite in the mold (Figure 3). However, it is noteworthy that with established dosage limits, all mixtures still achieved a consistency allowing for the preparation and molding of samples for testing. It was observed that the critical consistency, at which the workability of the mixture would be insufficient, was approximately 40 mm, and thus, this value was adopted as a worse value in the multi-criteria analysis. It was also noted that with an immersion depth of the measuring cone above 100 mm, there were no significant differences in the ease of laying and compacting composites, and therefore, this value was adopted as a better value in the analysis.

### 3.3. Compressive Strength

For the obtained compressive strength measurements results $f_{c}$, a GAM analysis was conducted. As a result, Equation (6) was obtained. For the obtained equation, goodness-of-fit checks were performed: MAPE was 9.3%, and $R^{2}$ was 0.80. The autocorrelation and partial autocorrelation functions of the residuals were within acceptable ranges, indicating that the obtained equation was a regression equation, and therefore, it could be utilized for further analysis.

(6) $$f_{c}=7.1648-0.0604PP-0.1379C+0.0006{PP}^{2}+0.0012PP\cdot C+0.0011C^{2}$$

**Figure 4:**
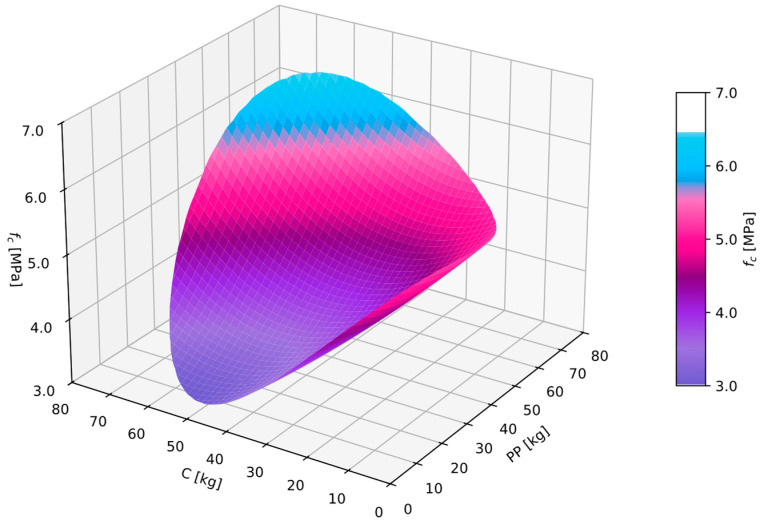
GAM function for compressive strength $f_{c}$ in dependence of perlite powder PP and cenosphares C.

It should be noted that with an increase in the dosage of perlite powder, the compressive strength significantly increased. The dosage of cenospheres also led to an increase in the compressive strength but to a lesser extent compared with the influence of perlite powder. A satisfactory compressive strength $f_{c}$ of 5 MPa was achieved with a dosage of perlite powder exceeding 35 kg/m^3^ (Figure 4).

### 3.4. Flexural Strength

**Figure 5:**
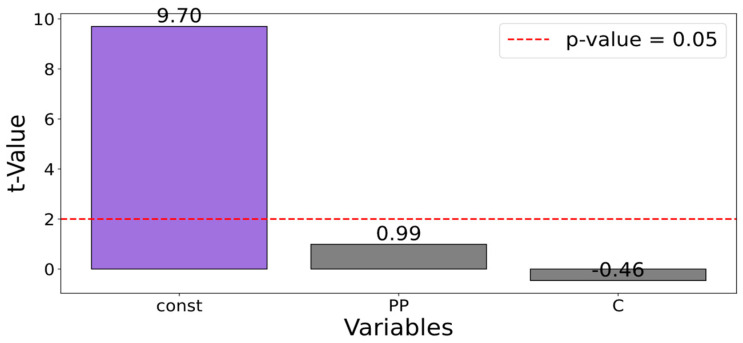
GAM function for flexural strength $f_{t}$, in dependence of perlite powder (*PP*) and cenosphares (*C*).

For the obtained flexural strength measurements results $f_{t}$, a GAM analysis was not conducted. The analysis for determining the regression equation was not performed, because the Pareto analysis coefficients of significance were below the critical value (Figure 5). Pareto analysis coefficients of significance quantified the relative importance of various factors by expressing their contributions as percentages of the total impact. These coefficients were used to identify the most significant causes. By calculating cumulative contributions, Pareto analysis helped prioritize resources and actions toward the most critical issues, improving efficiency and effectiveness in problem-solving. This indicated that the analyzed variables C and PP did not have a statistically significant impact on flexural strength. Therefore, modification with these components did not affect the flexural strength results. The discrepancies in the results were similar to the standard deviations within a single composition.

### 3.5. Thermal Conductivity Coefficient

For the obtained thermal conductivity coefficient λ results, a GAM analysis was conducted. As a result, Equation (7) was obtained. For the obtained equation, goodness-of-fit checks were performed: MAPE was 6.1%, and *R*^2^ was 0.70. The autocorrelation and partial autocorrelation functions of the residuals were within acceptable ranges, indicating that the obtained equation was a regression equation, and therefore, it could be utilized for further analysis.

(7) $$\lambda =0.3246-0.0025PP-0.003C+0.0000246{PP}^{2}+0.0000167PP\cdot C+0.0000287C^{2}$$

**Figure 6:**
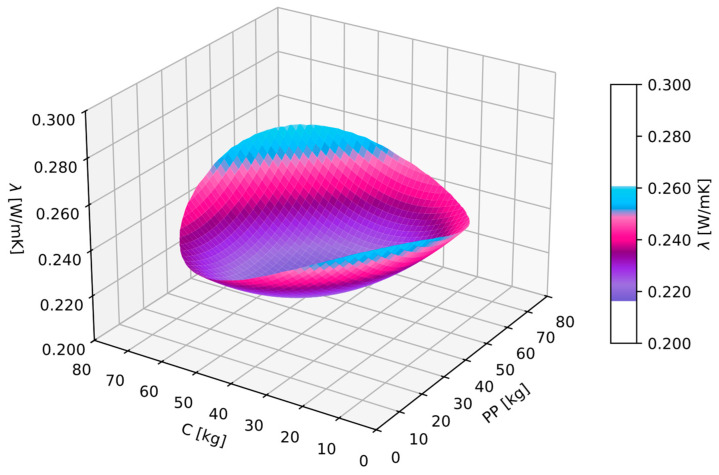
GAM function for thermal conductivity coefficient λ in dependence of perlite powder (*PP*) and cenosphares (*C*).

Analyzing the obtained function (Figure 6), it can be observed that the lowest thermal conductivity coefficient was achieved for composites with a dosage of perlite powder and cenospheres at the level of 35 kg/m^3^ of the mixture. It is worth noting that the modifications analyzed in the study did not significantly affect the thermal conductivity coefficient. The key parameter that influenced achieving good insulation for all composites was the preparation of mixtures with a significant proportion of perlite sand.

### 3.6. Utility Function

By obtaining regression equations for all analyzed dependent variables, it was possible to prepare a generalized utility function U. In this regard, GAM analysis was also utilized. Equation (8) was derived based on the previously obtained regression Equations (5)–(7). By assigning appropriate weights and considering values defined as the range of satisfactory technical properties—consistency, thermal conductivity, and compressive strength—it was possible to construct a comprehensive utility function. Equation (8) was obtained, and goodness-of-fit measures were examined: MAPE = 3.9% and $R^{2}$ = 0.84, along with autocorrelation and partial autocorrelation of residuals not exceeding permissible levels. Therefore, the obtained function could be considered a regression equation. In the analysis, better and worse values were adopted according to Table 4.

Better and worse values for consistency were established based on laboratory research and related conclusions regarding the workability of the mixture. The threshold values for flexural and compressive strength were adopted in accordance with the requirements for construction mortars. Weights were assigned to give the greatest importance to the fundamental characteristics required of the designed mortar, namely, compressive strength and thermal conductivity, while lower weight was assigned to workability. The weights adopted for the analysis of the generalized utility function were determined based on the authors’ decisions. These decisions were primarily driven by the intended use of the composite for the production of prefabricated cladding panels. As a result, compressive strength and thermal conductivity were considered equally important, reflecting their critical role in the composite’s performance. Additionally, due to the planned technological process, consistency was deemed less significant as adjustments such as compaction could be applied if necessary. It is also important to note that the increase in thermal insulation typically corresponded with a decrease in compressive strength. However, this relationship was not strongly correlated, as demonstrated in the preceding studies [26].

(8) $$U=0.3127+0.0059PP+0.0058C-0.00001{PP}^{2}-0.0001PP\cdot C-0.0000C^{2}$$

**Figure 7:**
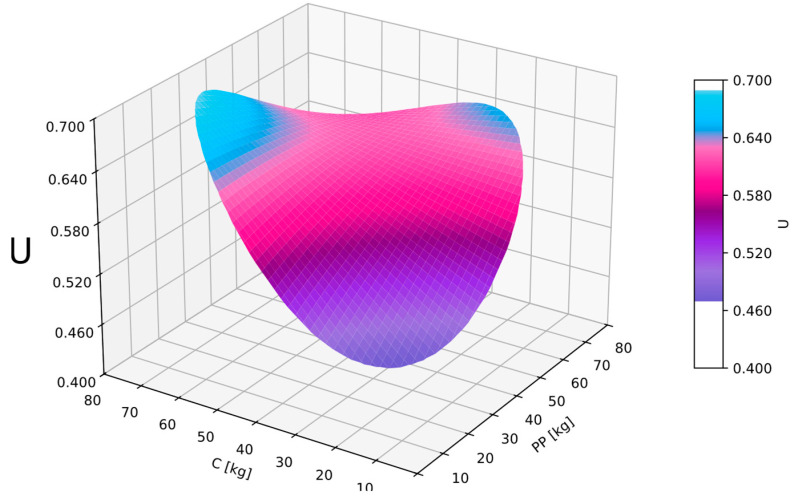
Utility function U in dependence of perlite powder (*PP*) and cenosphares (*C*).

Analyzing the generalized utility function U, it can be observed that the optimal composition was one in which the total dosage of perlite powder and cenospheres amounted to 75 kg per cubic meter of the mixture (Figure 7). For such a scenario, the value of the utility function exceeded 0.7.

## 4. Discussion and Conclusions

The ongoing climate changes necessitate decisive actions, especially in the field of construction. One action that could contribute to climate improvement is replacing cement with environmentally friendly alternatives, such as geopolymers obtained through the use of chemically activated fly ash waste. The aim of the conducted research was to assess the impacts of modifying the geopolymers with additives, potentially enhancing thermal insulation in their fundamental technical characteristics. The conducted studies indicated the following:

- The dosage of perlite dust and cenospheres significantly influenced the technical characteristics of geopolymers.
- Increasing the dosage of perlite dust and cenospheres did not unequivocally worsen the workability of the mixtures.
- Increasing the dosage of both perlite dust and cenospheres enhanced compressive and flexural strength.
- The lowest thermal conductivity coefficient of composites was achieved with the maximum dosage of cenospheres or perlite dust.
- There was a strong correlation between compressive and flexural strength and the consistency of the mixture—higher slump values corresponded to higher flexural and compressive strength.

The use of a generalized utility function identified optimal composite compositions where the sum of cenosphere and perlite dust dosage was around 75 kg/m^3^. This ensured good thermal insulation without significantly worsening the workability of the mixture and thereby not significantly compromising compressive and flexural strength. Excessive content of both additives or their absence adversely affected utility function values.

The findings of this study aligned well with the existing body of research on fly ash-based geopolymer composites, particularly in enhancing thermal insulation properties while maintaining adequate mechanical performance. The addition of modifiers such as perlite sand, waste perlite powder, and cenospheres demonstrated a significant influence on the thermal conductivity and compressive strength of these composites. These results were consistent with earlier studies indicating that introducing pores and voids in geopolymer matrices can effectively lower thermal conductivity values, which is crucial for thermal insulation applications [41,42,43].

The achieved thermal conductivity values, ranging between 0.18 and 0.24 W/(m·K), placed these composites within the classification of insulating materials, specifically aligning with Class III lightweight concretes as defined by RILEM [28]. These λ values were also comparable to previous research, where geopolymer foam concretes achieved thermal conductivity between 0.1 and 0.4 W/(m·K) [44]. Notably, this performance was attained with composite densities suitable for insulating purposes (typically between 500 and 700 kg/m^3^) and porosities ranging from 50% to 95% [45].

In terms of mechanical properties, the study’s findings confirmed that maintaining compressive strengths above 3.5 MPa, even with the addition of lightweight modifiers, was achievable, placing the composites in RILEM Class II for combined structural and insulating purposes [28]. However, the trade-off between porosity and strength remained a critical consideration as higher porosity tended to reduce the compressive strength, consistent with prior research where compressive strengths typically ranged between 3 and 5 MPa [46,47]. The identified optimal dosage of additives (approximately 75 kg per total mixture) highlights the importance of balancing thermal insulation improvements with mechanical integrity.

The aim of the conducted modification of geopolymers was to improve thermal insulation properties without compromising strength. The presented research highlights the significant potential of the analyzed components, especially when combined in their dosage. Further research encompassing chemical resistance analysis is necessary. A key outcome of this study is the confirmation that perlite powder can replace cenospheres as a cost-effective alternative without compromising material performance. This finding presents a sustainable use of industrial waste and potential cost savings. However, this would require a life cycle analysis considering the carbon footprint; the water footprint; and other environmental, technical, and social parameters.

## Figures and Tables

**Table 1:** Variable parameters of composite composition in the experiment plan.

Composition No.	Coded Variables		Actual Variables		
	$x_{1}$	$x_{2}$	PP [kg]	C [kg]	Experimental Plan
1	−1	−1	15.25	15.25	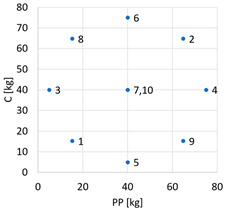
2	1	1	64.75	64.75	
3	−1.414	0	5.00	40.00	
4	1.414	0	75.00	40.00	
5	0	−1.414	40.00	5.00	
6	0	1.414	40.00	75.00	
7	0	0	40.00	40.00	
8	−1	1	15.25	64.75	
9	1	−1	64.75	15.25	
10	0	0	40.00	40.00	

Notes: $x_{1}$, $x_{2}$ are the coded variables; PP is the mass of perlite powder; C is the mass of cenospheres.

**Table 2:** Results of compressive strength, flexural Strength, and thermal conductivity tests.

Composition No.	Actual Variables		Consistency $\alpha_{con}$, mm	Flexural Strength $f_{t}$, MPa	Compressive Strength $f_{c}$, MPa	Thermal Conductivity λ, W/(m·K)
	PP, kg	*C*, kg				
1.	15.25	15.25	135	1.8 ± 0.3	4.6 ± 0.2	0.245 ± 0.020
2.	64.75	64.75	70	1.6 ± 0.1	5.6 ± 0.2	0.237 ± 0.014
3.	5.00	40.00	130	1.8 ± 0.1	4.3 ± 0.2	0.262 ± 0.006
4.	75.00	40.00	50	1.9 ± 0.1	6.1 ± 0.4	0.260 ± 0.005
5.	40.00	5.00	120	1.6 ± 0.2	5.0 ± 0.4	0.256 ± 0.011
6.	40.00	75.00	70	2.0 ± 0.1	6.7 ± 0.4	0.276 ± 0.038
7.	40.00	40.00	70	1.6 ± 0.3	3.5 ± 0.3	0.184 ± 0.010
8.	15.25	64.75	40	1.1 ± 0.1	1.9 ± 0.2	0.204 ± 0.005
9.	64.75	15.25	40	1.8 ± 0.2	5.3 ± 0.3	0.237 ± 0.024
10.	40.00	40.00	60	1.3 ± 0.1	4.3 ± 0.2	0.245 ± 0.004

Notes: PP is the mass of perlite powder; C is the mass of cenospheres.

**Table 3:** Results of basic statistical analysis.

Variable	Maximum	Minimum	Mean	Standard Deviation	Shapiro–Wilk Statistic	Shapiro–Wilk *p*-Value
Consistency $\alpha_{con}$ mm	135	40	78.5	36	0.85	0.052
Flexural strength $f_{c}$ MPa	2.2	1.1	1.6	0.3	0.95	0.152
Compressive strength $f_{t}$ MPa	7.2	1.6	4.8	1.3	0.95	0.052
Thermal conductivity λ W/(m·K)	0.28	0.18	0.24	0.03	0.90	0.237

**Table 4:** Utility function parameters.

Parameter	Consistency $\alpha_{con}$, mm	Compressive Strength $f_{c}$, MPa	Thermal Conductivity λ, W/(m·K)
Weight of the feature	0.2	0.4	0.4
Better value	100	10	0.20
Worse value	40	5	0.25

## Data Availability

The original contributions presented in the study are included in the article, further inquiries can be directed to the author.
